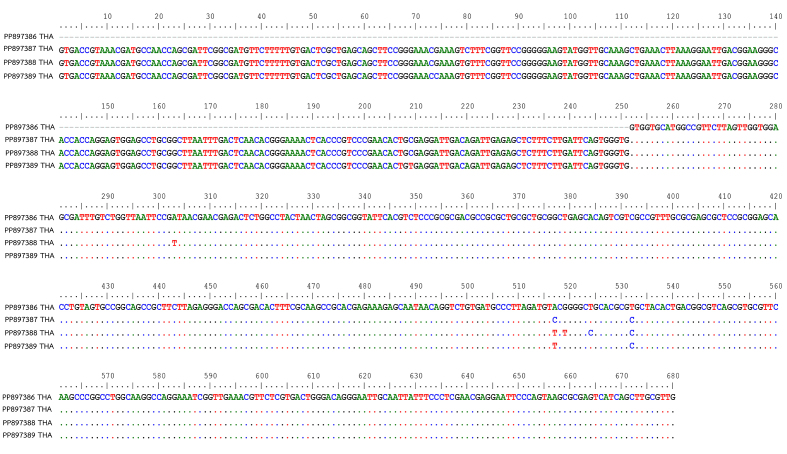# Molecular identification of *Trichuris* species in long-tailed macaques from Dong Ling Don Chao Pu Park and Kumphawapi Monkey Garden, Northeast Thailand: First report suggesting possible *Trichuris ovis* infection in non-human primates

**DOI:** 10.1016/j.ijppaw.2025.101063

**Published:** 2025-04-01

**Authors:** Issarapong Phosuk, Tongjit Thanchomnang, Julalak Banglua, Sakhone Laymanivong, Darunee Puangpronpitag, Jurairat Jongthawin

**Affiliations:** a Department of Public Health, Mahidol University, Amnatcharoen Campus, Amnat Charoen, 37000, Thailand; b Faculty of Medicine, Mahasarakham University, Maha Sarakham, 44000, Thailand; c Biomedical Science Research Unit, Mahasarakham University, Maha Sarakham, 44000, Thailand; d International and National Collaborative Network and Innovation for Community Health Development Research Unit, Mahasarakham University, Maha Sarakham, 44000, Thailand; e Unit of Water and Food Analysis, Division of Research, Department of Medical Science, Mahidol University, Amnatcharoen Campus, Amnat Charoen, 37000, Thailand; f Centre of Malariology, Parasitology and Entomology, Ministry of Health, P.O. Box 0100, Vientiane Capital, Laos

**Keywords:** Northeast Thailand, Long-tailed macaques, *T. trichiura*, *T. ovis*, 18S ribosomal RNA

## Abstract

This study aimed to molecularly identify *Trichuris* spp. in long-tailed macaques from two key habitats in Northeast Thailand: Dong Ling Don Chao Pu Park, Amnat Charoen Province, and Kumphawapi Monkey Garden, Udon Thani Province. Genomic DNA was extracted from 13 *Trichuris* spp. egg samples collected from 13 infected long-tailed macaques, and PCR amplification targeting partial sequences of the 18S rRNA gene and ITS2 region was performed for phylogenetic analysis. Of the 13 *Trichuris* spp. egg samples, the partial 18S rRNA gene was successfully amplified from six, while ITS2 amplification was unsuccessful. Phylogenetic analysis indicated that four specimens sequenced from Dong Ling Don Chao Pu Park were *T. trichiura*. In contrast, two specimens sequenced from Kumphawapi Monkey Garden clustered with the only confirmed *T. ovis* from goat, as well as unconfirmed *Trichuris* spp. from other ruminant hosts. These findings suggest that the *Trichuris* spp. in macaques are likely *T.ovis*; however, the evidence remains inconclusive. Therefore, accurate species identification in this region requires further molecular analysis using additional genetic markers. This study provides the first molecular identification of *T. trichiura* in long-tailed macaques from Northeast Thailand. Additionally, it is the first report suggesting the possible *T. ovis* infection in non-human primates. These findings highlight the potential for *Trichuris* spp. transmission across diverse host species, underscoring the need for enhanced surveillance of parasitic infections in wildlife and livestock, particularly in regions with close human-animal interactions. Continued molecular investigations are essential to elucidate *Trichuris* spp. transmission dynamics and zoonotic potential, aiding in public health risk mitigation.

## Highlights

• First molecular identification of *Trichuris trichiura* in long-tailed macaques from Northeast Thailand.
• First report suggesting the possible presence of *T. ovis* in non-human primates (NHPs).
• Findings indicate potential cross-species transmission of *Trichuris* between primates and ruminants, with possible zoonotic implications.

## Graphical abstract

**Figure:**
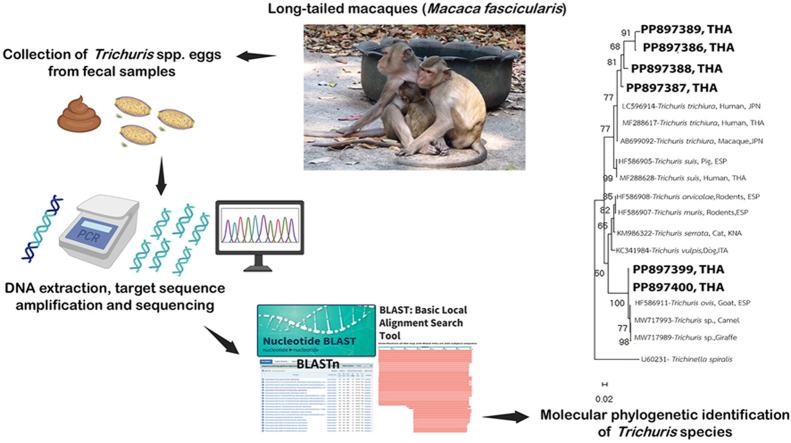

## 1 Introduction

Parasitic infections caused by intestinal helminths, particularly soil-transmitted nematodes such as *Trichuris* spp. (whipworms), represent a significant global health challenge and constitute a major component of neglected tropical diseases (NTDs). These parasites are associated with inflammation of the cecum and large intestine, bloody diarrhea, chronic iron deficiency anemia, and in severe cases, malnutrition and cognitive impairments. Infection occurs via the ingestion of soil or food with contaminating embryonated eggs, posing a significant risk to populations in endemic regions (Behniafar et al., 2024). Among the approximately 80 species of the genus *Trichuris* (Callejón et al., 2016), zoonotic species such as *Trichuris trichiura* (humans, non-human primates) (Liu et al., 2014; Phosuk et al., 2018; Sricharern et al., 2021), *T. suis* (pigs) (Kradin et al., 2006; Liu et al., 2014), and *T. vulpis* (dogs) (Márquez Navarro et al., 2012) have raised public health concerns, with *T. trichiura* being the most pathogenic to humans. Other species, including *T. ovis* (sheep), *T. skrjabini* (goat), and *T. muris* (mice), primarily infect animals (Liu et al., 2013). However, they may have indirect implications for human health through ecological overlap, adaptation, and potential cross-species transmission.

The long-tailed macaque (*Macaca fascicularis*), a widely distributed non-human primates (NHPs) species in Southeast Asia, including Thailand, serves as a critical host and reservoir for gastrointestinal (GI) helminths. These include *Trichuris* spp., *Strongyloides* spp., hookworms, and other parasitic species with zoonotic potential (Buppan et al., 2017; Pumipuntu, 2018; Schurer et al., 2019; Thanchomnang et al., 2019; Damrongsukij et al., 2021; Sricharern et al., 2021; Phosuk et al., 2024). In Thailand, long-tailed macaques are found at over 91 sites (Malaivijitnond and Hamada, 2008; Schurer et al., 2019), often in close proximity to human populations due to habitat encroachment and increasing human-macaque interactions. This overlap creates opportunities for the zoonotic transmission of GI parasites, posing significant public health risks, particularly in areas frequented by tourists or where macaques inhabit urban and peri-urban environments (Thanchomnang et al., 2017).

*Trichuris* spp. infections in long-tailed macaques in Thailand have been documented, with reported prevalence rates ranging from 1.5 % to 27 % (Buppan et al., 2017; Pumipuntu, 2018; Schurer et al., 2019; Thanchomnang et al., 2019; Damrongsukij et al., 2021; Sricharern et al., 2021; Phosuk et al., 2024). However, accurate species-level identification remains challenging due to the morphological similarities of *Trichuris* eggs (García-Sánchez et al., 2020), which limit the reliability of conventional microscopic diagnostic methods. Molecular techniques, such as DNA-based analysis, are crucial for precise species identification, elucidating parasite diversity, and assessing potential zoonotic risks (Callejón et al., 2013; Thanchomnang et al., 2017; Phosuk et al., 2018). Despite their utility, molecular approaches in this context have been underutilized, with only one published study to date confirming the molecular identification of *T. trichiura* in long-tailed macaques in Lopburi, Central Thailand, using internal transcribed spacer 1 (ITS1) sequence (Sricharern et al., 2021). Known as the human whipworm, *T. trichiura* is a significant parasitic pathogen, causing asymptomatic or mild infections in light cases but leading to severe health issues in heavy infestations, including abdominal discomfort, bloody diarrhea, anemia, malnutrition, bowel obstruction, and colon perforation. In children, *T. trichiura* can result in Trichuris Dysentery Syndrome (TDS), characterized by anemia, growth retardation, and cognitive impairment (Khuroo et al., 2010). The identification of *T. trichiura* in long tailed macaques, as well as its documentation in other non-human primates such as baboons, squirrel monkeys, African green monkeys, and woolly monkeys (Eo et al., 2019), underscores its broad host range and potential zoonotic risk. Non-human primates, often living in close proximity to human populations in regions like Thailand, could serve as reservoirs or vectors for transmission, particularly in areas where human-primate interactions are frequent.

This study aims to molecularly characterize *Trichuris* spp. infecting long-tailed macaques in Northeast Thailand by analyzing partial sequences of the 18S ribosomal RNA (18S rRNA) and the internal transcribed spacer 2 (ITS2) regions. The research focuses on two key locations—Kumphawapi Monkey Garden in Udon Thani Province and Dong Ling Don Chao Pu Park in Amnat Charoen Province—where frequent human-macaque interactions heighten the risk of zoonotic transmission. By identifying the *Trichuris* species present and assessing their genetic diversity, this study seeks to enhance understanding of their epidemiology, host specificity, and potential for cross-species transmission. The findings will support improved surveillance and control measures, ultimately contributing to the protection of both human and animal health, particularly in areas with close human-primate interactions.

## 2 Materials and methods

### 2.1 Study area and sample collection

Between March and April 2022, thirteen *Trichuris* spp. egg samples, each containing approximately 200 eggs, were recovered from thirteen infected long-tailed macaques at two well-known locations in Northeast Thailand. Seven samples were obtained from Dong Ling Don Chao Pu Park in the Phana District of Amnat Charoen Province, while six were collected from Kumphawapi Monkey Garden in the Kumphawapi District of Udon Thani Province. Both locations are recognized for their free-ranging long-tailed macaque populations, which frequently interact with humans and, to varying degrees, with domestic animals. For fecal sample collection, feces were promptly collected from the ground immediately after defecation. Samples were gathered directly from areas where macaques actively defecated, rather than from soil alone, to minimize environmental contamination. Only macaques previously confirmed to be *Trichuris*-positive through parasitological screening were selected for molecular analysis in this study. Fecal samples were processed using the JK-Parasite Trap® (J. SILP Engineering, Thailand), following the manufacturer's instructions. This device efficiently isolates parasitic eggs from fecal samples using a combination of filtration and sedimentation techniques, effectively separating parasite eggs from fecal debris to ensure a higher concentration of eggs for microscopic examination and molecular analysis. A demonstration of the device and its methodology is available in a video produced by the Faculty of Medicine, Khon Kaen University, Thailand (https://www.youtube.com/watch?v=KI0yvLRLKfk). The egg samples were collected, preserved in 70 % ethanol, and stored at −80 °C prior to DNA extraction.

### 2.2 DNA extraction, PCR amplification and DNA sequencing

Genomic DNA was extracted from thirteen *Trichuris* spp. egg samples, each containing approximately 200 eggs using the GF-1 tissue kit (Vivantis Technologies Sdn. Bhd., Malaysia), according to the manufacturer's instructions. A 727 bp fragment of the 18S ribosomal RNA (18S rRNA) gene was amplified using the forward primer 18S965F and reverse primer 18S1573R, which are specific to the *Trichuris* genus, as described by Guardone et al. (2013). Each PCR reaction was performed in a 25-μL reaction volume consisting of 2X vired taq (Vivantis Technologies Sdn. Bhd., Malaysia), 2 mM of MgCl_2_, 2 μM of each primer, and 5 μL of DNA template. The DNA template was initially denatured at 94 °C for 5 min. The amplification procedure included 35 cycles at 95 °C for 30 s (denaturation), 59 °C for 30 s (annealing), and 72 °C for 30 s (extension), with a final extension at 72 °C for 10 min. The DNA of *T. trichiura* was used as positive control and ultrapure water as non-template control. For the ITS2 region amplification (325 bp), the primers, PCR reaction preparation, and amplification procedure were performed as previously described (Phosuk et al., 2018). The PCR was carried out using a GeneAmp® PCR System 9700 (Applied Biosystems, Foster City, CA). Amplified products were run on a 1 % agarose gel electrophoresis and visualized with a UV light image system (E-Box, Vilber, France). A positive band was purified and sequenced at a commercial facility (1st Base, Malaysia). DNA fragments were sequenced in both directions using the same primers employed in the PCR.

### 2.3 Sequence analysis and phylogenetic tree construction

**Fig. 1:**
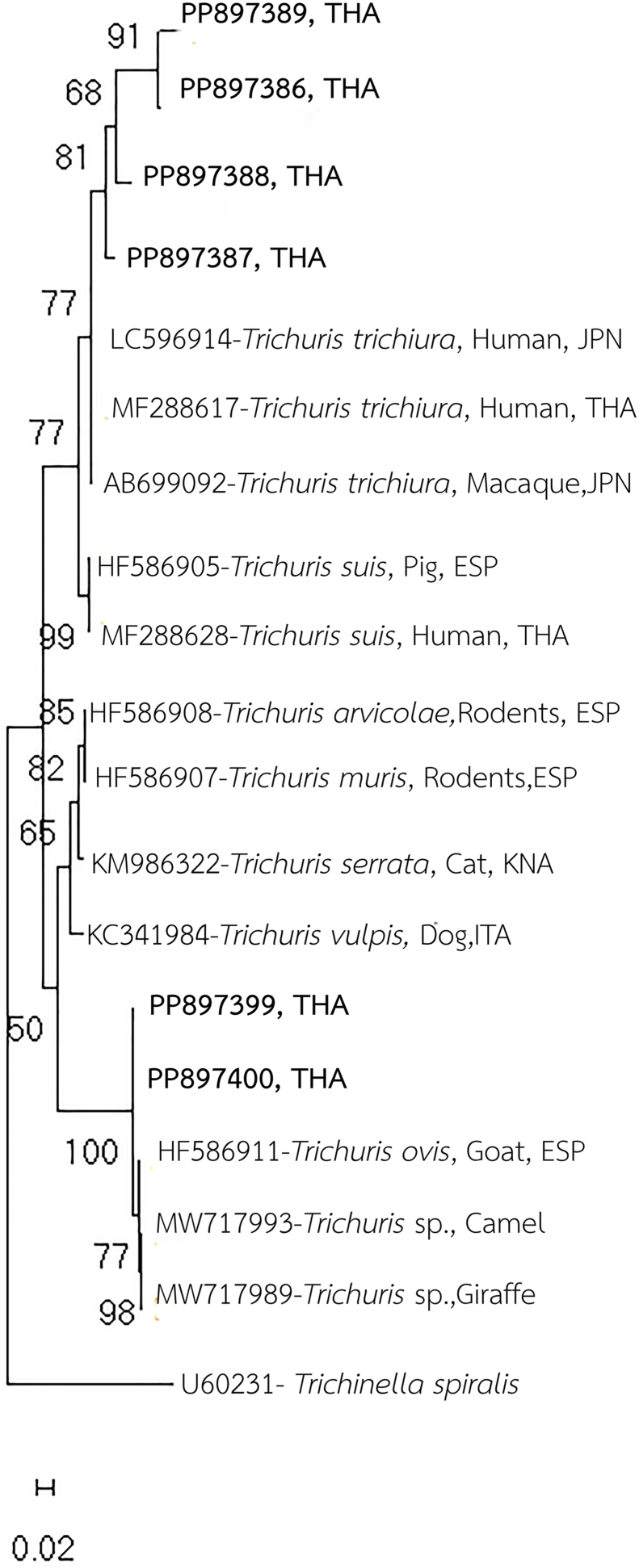
Phylogenetic relationships among *Trichuris* spp. Reference sequences from various *Trichuris* spp. and *Trichinella spiralis* (used as the outgroup) were retrieved from GenBank and are annotated with their accession numbers and ISO 3166-1 alpha-3 country codes. The partial 18S rRNA sequences of *Trichuris* spp. generated in this study are highlighted in bold. Bootstrap support values (as percentages from 1000 replicates) are shown at each node of the tree. Scale bars represent the number of substitutions per nucleotide position.

To determine the species, the new partial 18S rRNA gene sequences from *Trichuri*s spp. egg samples were analyzed using the BLASTN search tool (National Center for Biotechnology Information [NCBI], Bethesda, MD, USA). These sequences were aligned with reference sequences from the GenBank using the BioEdit Sequence Alignment Editor (Hall, 1999). The available alignment lengths after sequencing and primer trimming are summarized in Table 1. Phylogenetic relationships among *Trichuris* spp. were constructed using the maximum likelihood method based on the Tamura 3-parameter model, implemented in MEGA version 11 (Tamura et al., 2021). The selected dataset and support for clusters in each tree were calculated using 1000 bootstrap replications. Detailed nucleotide sequence information is provided in Fig. 1. Genetic distance values between the new partial 18S rRNA sequences of *Trichuris* spp. and reference sequences were also calculated using the Tamura-Nei model. (Tamura and Nei, 1993).

**Table 1:** Sample details, including collection locations, nucleotide lengths obtained after sequencing and primer trimming, and GenBank accession numbers of the new partial 18S rRNA sequences.

Sample No.	Locations	Nucleotide length analyzed (18S rRNA)	GenBank Accession No.
1	1. Dong Ling Don Chao Pu Park,	429 bp	**PP897386**
2	Amnat Charoen province	680 bp	**PP897387**
3	(15°40′13.9″N 104°51′26.0″E)	680 bp	**PP897388**
4		680 bp	**PP897389**


5	2. Kumphawapi Monkey Garden,	508 bp	**PP897399**
6	Udon Thani province	416 bp	**PP897400**
	(17°06′ 41.8″N, 103°01′ 03.4″E)

## 3 Results

### 3.1 Molecular phylogenetic identification of *Trichuris* species in long-tailed macaques

Of the 13 *Trichuris* spp. egg samples collected from thirteen infected long-tailed macaques, a 727-bp PCR product corresponding to a partial 18S rRNA gene was successfully amplified from the genomic DNA of six samples. After sequencing and primer trimming, the nucleotide lengths available for analysis were as follows: sample 1: 429 bp; sample 2: 680 bp; sample 3: 680 bp; sample 4: 680 bp; sample 5: 508 bp; and sample 6: 416 bp. However, attempts to amplify the ITS2 region were unsuccessful (Table 1). Sequence alignment of the four new *Trichuris* partial 18S rRNA sequences from long-tailed macaques at Dong Ling Don Chao Pu Park revealed five variable sites among the sequences (Supplementary Fig. S1). Phylogenetic analysis placed all four sequences within the same clade as published *T. trichiura* sequences from various hosts, including humans from Japan and Thailand (GenBank accession numbers: LC596914, MF288617) and *Macaca fuscata* from Japan (AB699092) (Fig. 1). BLAST-N analysis further confirmed a sequence similarity of 98 %–99.78 %, supporting the identification of the *Trichuris* spp. infecting long-tailed macaques at Dong Ling Don Chao Pu Park, Amnat Charoen Province, in Northeast Thailand, as *T. trichiura.*

**Fig. 2:**
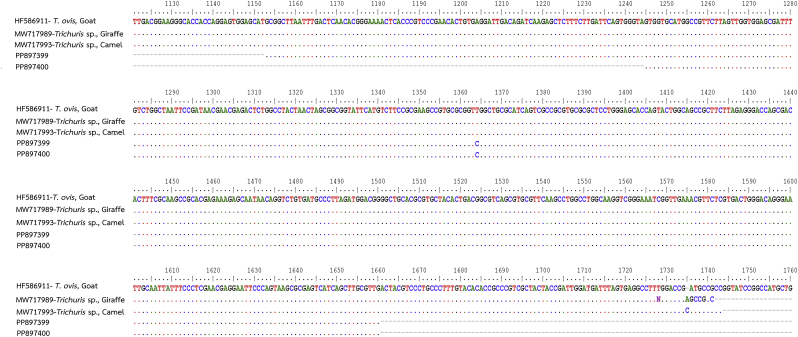
Sequence alignment of two new partial 18S rRNA sequences of *Trichuris* spp. from long-tailed macaques in Kumphawapi Monkey Garden (PP897399 and PP897400) with the published partial 18S rRNA sequences of *T. ovis* and unconfirmed *Trichuris* spp. from GenBank.

Additionally, two partial 18S rRNA sequences of *Trichuris* spp. egg samples from long-tailed macaques at Kumphawapi Monkey Garden were analyzed, with no DNA variation detected between the samples (Fig. 2). Phylogenetic analysis placed these specimens sequenced in the same clade as *T. ovis* from *Capra hircus* (goat) in Spain (HF586911) (Fig. 1), with a 99.78 % similarity based on BLAST-N analysis. Furthermore, the sequences also exhibited 99.78 % similarity to unconfirmed *Trichuris* spp. from a camel (MW717993) and a giraffe (MW717989). These findings suggest that the *Trichuris* spp. identified in long-tailed macaques at Kumphawapi Monkey Garden, Kumphawapi District, Udon Thani Province, Northeast Thailand, are likely *T. ovis.*

The genetic relationships among six *Trichuris* spp. egg samples and related sequences were analyzed based on partial 18S rRNA gene sequences, using genetic distance values. (Table 2). The results indicate that the four *Trichuris* spp. egg samples from long-tailed macaques in Dong Ling Don Chao Pu Park share genetic distances ranging from 0.005 to 0.020 with the reference *T. trichiura* sequence (AB699092) from GenBank, indicating a close genetic relationship. Similarly, the two *Trichuris* spp. egg samples from long-tailed macaques in Kumphawapi Monkey Garden, along with the reference *T. ovis* sequence (HF586911) and unconfirmed *Trichuris* spp. from a camel (MW717993) and a giraffe (MW717989), exhibit genetic distances ranging from 0.000 to 0.002, further supporting their close genetic relatedness. However, the genetic distance between the *Trichuris* spp. egg samples from Dong Ling Don Chao Pu Park and those from Kumphawapi Monkey Garden ranges from 0.052 to 0.067, indicating a clear genetic distinction. These findings suggest that the *Trichuris* spp. egg samples from the two locations belong to different species.

**Table 2:** Genetic distance values based on partial 18S rRNA sequences among *Trichuris* spp. collected. from long-tailed macaques in Dong Ling Don Chao Pu Park and Kumphawapi Monkey Garden, compared with reference sequences from GenBank.

		1	2	3	4	5	6	7	8	9	10
1	**PP897399 - *Trichuris* spp.**										
2	**PP897400 - *Trichuris* spp.**	0.000									
3	**PP897386 - *T. trichiura***	0.052	0.052								
4	**PP897387***-****T. trichiura***	0.056	0.058	0.005							
5	**PP897388 - *T. trichiura***	0.063	0.067	0.012	0.007						
6	**PP897389 - *T. trichiura***	0.054	0.058	0.005	0.006	0.007					
7	AB699092-*T. trichiura -Macaca fuscata*	0.040	0.050	0.005	0.017	0.020	0.012				
8	HF586911-*T. ovis -Capra hircus (goat)*	0.002	0.002	0.055	0.052	0.056	0.047	1.388			
9	MW717989-*Trichuris* spp*.- Giraffe*	0.002	0.002	0.055	0.052	0.056	0.047	1.143	0.002		
10	MW717993-*Trichuris* spp*.- Camel*	0.002	0.002	0.055	0.052	0.056	0.047	1.139	0.001	0.001	

All six newly identified 18S rRNA sequences of *Trichuris* spp. from long-tailed macaques in this study have been deposited in the GenBank under accession numbers PP897386–PP897389 and PP897399–PP897400 (Table 1).

## 4 Discussion

In Thailand, several studies have reported *Trichuris* spp. infections in long-tailed macaques; however, species-level confirmation remains extremely limited. Accurate identification of *Trichuris* species is critical for understanding their epidemiology and potential zoonotic impact. DNA markers have proven to be powerful tools for species identification, with commonly used markers including ribosomal repeat region genes such as the internal transcribed spacer regions (ITS1 and ITS2), the 18S rRNA gene, and mitochondrial DNA (mtDNA) markers like the cox1 gene (Callejón et al., 2013; Liu et al., 2013; Phosuk et al., 2018; Sricharern et al., 2021; Moré et al., 2024). The 18S rRNA gene stands out as particularly advantageous for studying the phylogenetic relationships among *Trichuris* spp. It is less prone to alignment ambiguities compared to ITS sequences and provides robust phylogenetic resolution, even when ambiguous sites are excluded (Callejón et al., 2013). By contrast, the cox1 gene, while useful for distinguishing closely related species, offers limited utility for deeper phylogenetic relationships due to homoplasy caused by multiple substitutions at deeper nodes (Mas-Coma and Bargues, 2009). Given these advantages, our study utilized the partial 18S rRNA gene and also ITS2 region to identify *Trichuris* spp. infecting long-tailed macaques. However, attempts to amplify the ITS2 region were unsuccessful.

The unsuccessful amplification of the ITS2 region in our study contrasts with the only previous molecular investigation of *Trichuris* spp. in long-tailed macaques from Lopburi, Central Thailand, which successfully amplified the ITS1 region to confirm the presence of *T. trichiura* (Sricharern et al., 2021). That study reported a low detection rate, with only 3 out of 200 samples testing positive via nested PCR. The similarly low PCR success rate observed in both this and previous studies suggests challenges in amplifying the ITS regions from *Trichuris* eggs, potentially due to low DNA quantity, DNA degradation, or inhibitory substances in fecal samples. In contrast, our previous research successfully amplified the ITS2 region to identify *Trichuris* spp. in human hosts (Phosuk et al., 2018), suggesting that the efficacy of ITS markers may vary depending on host species or sample conditions. These findings highlight the need for further optimization of ITS-based detection methods for *Trichuris* in non-human primates.

Our study, based on partial 18S rRNA gene analysis, provides the first molecular confirmation of *T. trichiura* in long-tailed macaques from Dong Ling Don Chao Pu Park, Amnat Charoen province, Northeast Thailand. This finding corroborates prior evidence of *T. trichiura* infections in long-tailed macaques from Lopburi, Central Thailand, identified using ITS1 sequences (Sricharern et al., 2021).

Analysis of the partial 18S rRNA gene from *Trichuris* egg samples collected from long-tailed macaques at Kumphawapi Monkey Garden revealed a 99.78 % similarity to the only confirmed *T. ovis* sequence from *Capra hircus* (goat) (HF586911). However, the same level of similarity was also observed with unconfirmed *Trichuris* spp. sequences from a camel (MW717993) and a giraffe (MW717989). This high genetic similarity suggests that the *Trichuris* spp. in macaques may be *T. ovis*; however, the evidence remains inconclusive. Further molecular analyses, including additional genetic markers such as ITS1, ITS2, and cox1, or whole-genome sequencing, are required to confirm the definitive species of *Trichuris* in long-tailed macaques in this area.

*Trichuris ovis* is typically found in ruminants such as sheep, goats, and cattle. While infections in these hosts are often asymptomatic, severe cases can lead to intestinal damage, anemia, dehydration, and even mortality (Bhat et al., 2025). The detection of *T. ovis* or a closely related species in long-tailed macaques suggests that ecological interactions may facilitate its transmission.

Kumphawapi Monkey Garden, located in an urbanized area surrounded by temples and residential zones, supports a large population of free-ranging long-tailed macaques. These macaques frequently interact with humans, receiving food from visitors and monks, and are in close contact with domestic animals, including dogs, cats, and livestock particularly cattle. Such interactions increase the risk of environmental contamination and parasite spillover, potentially leading to cross-species transmission, aligning with the findings of this study. Although *T. ovis* is not currently recognized as a zoonotic pathogen, its presence in primates raises concerns about potential host expansion and adaptation. This highlights the need for further investigation into its transmission dynamics, host specificity, and potential implications for both animal and public health.

## 5 Conclusions

This study provides the first molecular identification of *T. trichiura* in long-tailed macaques from Northeast Thailand. Additionally, it is the first report suggesting the possible presence of *T. ovis* in non-human primates. Our findings enhance the understanding of the host range and transmission dynamics of *Trichuris* spp., highlighting the need for further research to confirm the presence of *T. ovis* and evaluate its potential zoonotic risks in non-human primates. These results underscore the value of molecular tools in parasite identification and emphasize the importance of integrated surveillance programs that monitor wildlife, livestock, and human health. Continued molecular investigations are essential for deepening our understanding of *Trichuris* transmission dynamics, host specificity, and zoonotic potential, ultimately informing public health strategies.

## CRediT authorship contribution statement

**Issarapong Phosuk:** Writing – review & editing, Writing – original draft, Project administration, Methodology, Formal analysis, Data curation. **Tongjit Thanchomnang:** Writing – review & editing, Validation, Supervision. **Julalak Banglua:** Writing – review & editing, Validation, Methodology, Data curation. **Sakhone Laymanivong:** Writing – review & editing, Validation, Supervision. **Darunee Puangpronpitag:** Writing – review & editing, Validation, Supervision. **Jurairat Jongthawin:** Writing – review & editing, Writing – original draft, Methodology, Investigation, Funding acquisition, Formal analysis, Conceptualization.

## Ethics statement

This study was conducted with the approval of the Mahidol University Institute Animal Care and Use Committee (MU-IACUC), Nakhon Pathom, Thailand (COA No. MU-IACUC2021/007).

## Availability of data and materials

Nucleotide sequence data reported in this paper are available in GenBank under accession numbers PP897386–PP897389 and PP897399–PP897400.

## Funding

This research project was financially supported by 10.13039/501100007288Mahasarakham University, Thailand.

## Declaration of conflict of interest

We, the authors of the research article titled: **Molecular identification of *Trichuris* species in long-tailed macaques from Dong Ling Don Chao Pu Park and Kumphawapi Monkey Garden, Northeast Thailand: First report suggesting possible *Trichuris ovis* infection in non-human primates**, authored by Issarapong Phosuk, Tongjit Thanchomnang, Julalak Banglua, Sakhone Laymanivong, Darunee Puangpronpitag, and Jurairat Jongthawin*,* hereby declare that we have no conflicts of interest to disclose. We affirm that there are no financial, personal, or professional affiliations that could have influenced the research, analysis, or conclusions presented in this study. Additionally, no funding bodies or external organizations had any role in study design, data collection, interpretation, manuscript preparation, or decision to submit the article for publication.

## Figures and Tables

**figs1:**